# Association between *KRAS* gene mutation and clinicopathological factors in patients with metastatic colorectal cancer

**DOI:** 10.1590/0102-672020260000048e1977

**Published:** 2026-10-05

**Authors:** Eduardo Toaza, Eva Valadares Soares, Eduardo Cambruzzi

**Affiliations:** 1 Grupo Hospitalar Conceição, Deparment of Pathology – Porto Alegre (RS), Brazil.

**Keywords:** Adenocarcinoma, Pathology, Prognosis, Carcinogenesis, Biomarkers, Tumor, Adenocarcinoma, Patologia, Prognóstico, Carcinogênese, Biomarcadores Tumorais

## Abstract

**Background:**

Colorectal cancer is a global health problem with high incidence and mortality rates, frequently diagnosed at metastatic stages. Mutations in the *KRAS* gene play a key role in colorectal cancer progression, significantly influencing treatment resistance and prognosis.

**Aims:**

This study investigated the association between clinicopathological characteristics and *KRAS* mutations in patients with metastatic colorectal cancer.

**Methods:**

This retrospective study evaluated medical records of patients with metastatic colorectal carcinoma who underwent *KRAS* gene mutation testing. A total of 96 patients treated in 2024 were included in the analysis.

**Results:**

*KRAS* mutations were identified in 63.54% of patients, predominantly in codon 12 (70.49%) and codon 13 (14.75%). No significant associations were found between *KRAS* mutation status and factors such as sex, age, or tumor location. Nevertheless, *KRAS* mutations were more frequently observed in patients with left-sided colon tumors, contrasting with the findings described in the literature. *KRAS* mutations were associated with poorer prognosis, higher rates of metastases (particularly to the liver), and higher rates of resistance to conventional treatments, such as chemotherapy.

**Conclusions:**

This study highlights the importance of *KRAS* mutation testing to support personalized, more effective treatment strategies to improve the prognosis of patients with metastatic colorectal cancer.

## ARTICLE HIGHLIGHTS

- KRAS gene mutations were frequently identified in metastatic colorectal cancer.
- Mutations in codons 12 and 13 predominated and were associated with tumor progression.
- KRAS mutations were associated with a poorer prognosis and a higher incidence of liver metastases.
- No significant association was found between KRAS mutation status and sex, age, or tumor location.

## Summary

**CENTRAL MESSAGE:**

Colorectal cancer is caused by mutations affecting oncogenes, tumor suppressor genes, and DNA repair pathways. The high mortality associated with colorectal adenocarcinoma is largely attributable to the development of metastases, with the liver being the most common site of dissemination. Epidemiological studies indicate that approximately 20 to 30% of patients with colorectal cancer present with liver metastases at the time of diagnosis. However, genetic variations associated with disease progression contribute to differences in therapeutic response and prognosis among patients with the same disease stage. Therefore, identifying more sensitive and specific molecular markers is essential for more effective tumor progression monitoring and improved prediction of clinical outcomes.

**PERSPECTIVES:**

*KRAS* mutation was identified in more than half of patients, being notably higher than the expected range. Contrary to the well-established association between KRAS mutations and right-sided colorectal tumors, patients with mutated KRAS in our cohort predominantly presented with left-sided tumors, particularly in the sigmoid colon. This unexpected finding may reflect genetic and environmental characteristics of the Gaucho population or interactions with other molecular drivers, warranting validation in independent cohorts. KRAS mutations are generally associated with a poorer prognosis, including increased metastatic potential and reduced response to conventional therapies, ultimately resulting in worse overall survival and disease-free survival.

## VISUAL ABSTRACT

**Figure:**
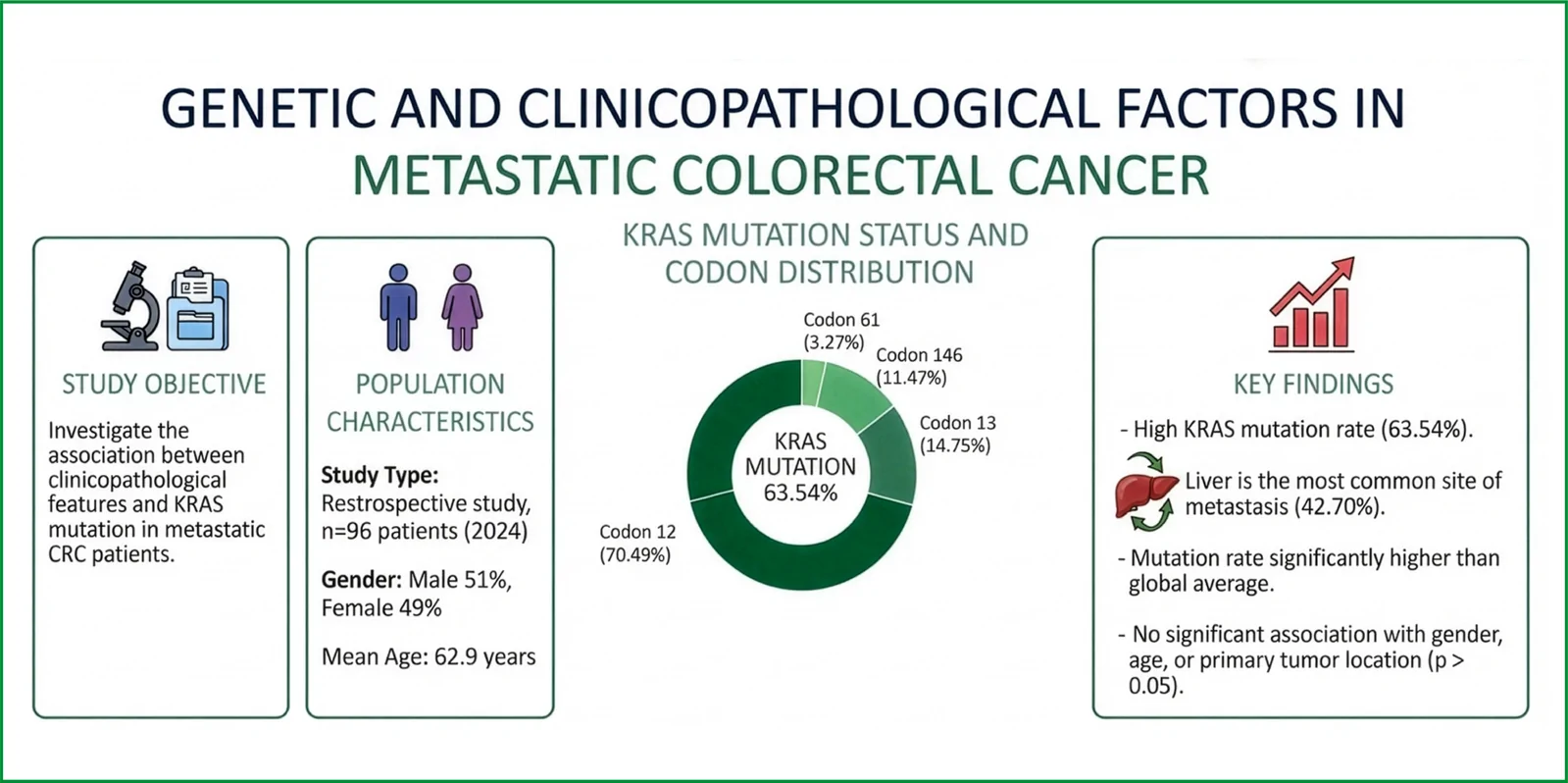

## RESUMO

**Racional:**

O câncer colorretal (CCR) é um problema de saúde global, com alta taxa de incidência e mortalidade, sendo frequentemente diagnosticado nos estágios metastáticos. As mutações no gene *KRAS* desempenham um papel fundamental na progressão do CCR, especialmente em relação ao prognóstico e à resistência ao tratamento.

**Objetivos:**

Este estudo teve como objetivo investigar a associação entre as características clinicopatológicas e a presença de mutações no gene *KRAS* em pacientes com câncer colorretal metastático.

**Métodos:**

Este estudo retrospectivo avaliou os prontuários médicos de pacientes com carcinoma colorretal metastático que foram submetidos à pesquisa da mutação do gene *KRAS*. Foram incluídos 96 pacientes na análise, tratados durante o ano de 2024.

**Resultados:**

A pesquisa revelou que 63,54% dos pacientes apresentaram mutação *KRAS*, predominando no códon 12 (70,49%) e no códon 13 (14,75%). Não houve associação significativa entre a mutação *KRAS* e fatores como sexo, idade e localização do tumor, embora a presença de mutações *KRAS* tenha sido mais comum em pacientes com tumores no cólon esquerdo, ao contrário do que é descrito na literatura. A mutação *KRAS* foi associada a um pior prognóstico, maior taxa de metástases, especialmente no fígado, e resistência a tratamentos convencionais, como a quimioterapia.

**Conclusões:**

Este estudo destaca a importância da análise genética para um tratamento personalizado e mais eficaz, contribuindo para a melhora do prognóstico dos pacientes com CCR metastático.

## INTRODUCTION

Colorectal cancer (CRC) is one of the most common malignancies worldwide, representing a major global health challenge. In the United States, approximately 153,000 new cases of CRC are diagnosed annually^29^. In Brazil, an estimated 45,630 new cases of CRC are expected each year during the 2023–2025 triennium^12^. CRC is the third most common cancer among men and the second among women. Established risk factors include a sedentary lifestyle, high consumption of red meat, alcohol use, and obesity^14^. Additional risk factors are related to genetic and hereditary conditions^7,21,28,29^.

The high mortality associated with colorectal adenocarcinoma is largely attributable to the development of metastases, with the liver being the most common site of dissemination. Epidemiological studies indicate that approximately 20 to 30% of patients with CRC present with liver metastases at the time of diagnosis^2,7,10,11,19^. Pathological staging using the Tumor, Lymph Nodes, and Metastasis (TNM) classification remains the primary prognostic factor guiding treatment decisions^2^. However, genetic alterations associated with disease progression contribute to differences in therapeutic response and prognosis among patients with the same disease stage^27^. Therefore, the identification of more sensitive and specific molecular markers is essential for improving tumor progression monitoring and predicting clinical outcomes^2,4,5,21^.

CRC is caused by mutations affecting oncogenes, tumor suppressor genes, and DNA repair pathways. Three major molecular pathways have been described in colorectal carcinogenesis: chromosomal instability, microsatellite instability, and the CpG island methylator phenotype. The chromosomal instability pathway, also known as the classical adenoma–carcinoma sequence, is characterized by gain-of-function mutations that disrupt the balance between oncogenes and tumor suppressor genes. Common mutations involve the *APC (*adenomatous polyposis coli), *KRAS (*Kirsten rat sarcoma viral oncogene homolog), and *TP53 (Treponema pallidum* 33 protein) genes^17^. Approximately 40 to 52% of CRC cases harbor mutations in the *KRAS* gene^30^. *KRAS* belongs to the *RAS* gene family and participates in signaling pathways that regulate cell proliferation, differentiation, and survival^22^. The gene comprises four coding exons and one non-coding exon, with mutations occurring most frequently in codons 12 and 13 of exon 2^17,21,24,25,30^.

This study evaluated a cohort of patients with metastatic CRC to investigate the association between *KRAS* mutation status, clinicopathological characteristics, and survival outcomes.

## METHODS

This cross-sectional study conducted a retrospective analysis of the electronic medical records of 96 patients with metastatic CRC treated at Hospital Nossa Senhora da Conceição, Porto Alegre, Brazil, in 2024. All patients had undergone molecular testing for *KRAS* mutations. The study was approved by the local Research Ethics Committee (CAAE No. 95038625.4.0000.5530).

Eligible participants included patients with colorectal adenocarcinoma who had metastatic disease at diagnosis or developed metastases during follow-up. All patients treated between January 1, 2024, and December 31, 2024, were included. Patients with insufficient tumor material in the paraffin block for molecular analysis were excluded. The following clinicopathological variables were analyzed: primary tumor size, tumor location, metastatic site, pathological stage, baseline carcinoembryonic antigen (CEA) level, treatment received, clinical status, and *KRAS* mutation status.

Data obtained from the Department of Pathology were entered into a Microsoft Excel spreadsheet and analyzed using JASP (Jeffreys's Amazing Statistics Program) software. Descriptive and comparative analyses were performed. Categorical variables are presented as absolute numbers and percentages, whereas continuous variables are expressed as measures of central tendency (mean and median) and dispersion (standard deviation). Disease-free survival was analyzed using Kaplan–Meier curves. All ethical principles governing human research were observed. Participant confidentiality was maintained by replacing personal identifiers with coded data, thereby ensuring anonymity throughout the study.

## RESULTS

The study included 96 patients with metastatic CRC, comprising 49 (51%) men and 47 (49%) women. Patient age ranged from 35 to 95 years, with a mean age of 62.96 years and a median age of 64.5 years.

*KRAS* mutations were identified in 61 (63.54%) patients. Among these, 43 (70.49%) involved codon 12, nine (14.75%) involved codon 13, seven (11.47%) involved codon 146, and two (3.27%) involved codon 61.

Mutations in the *BRAF* and *NRAS* genes were detected in seven (7.29%) and four (4.16%) patients, respectively. No patient harbored concurrent *KRAS* and *BRAF* or *NRAS* mutations. At the time of data collection, 27 (28.12%) patients had died, of whom 20 (74.07%) carried a *KRAS* mutation (Figure 1).

**Figure 1:**
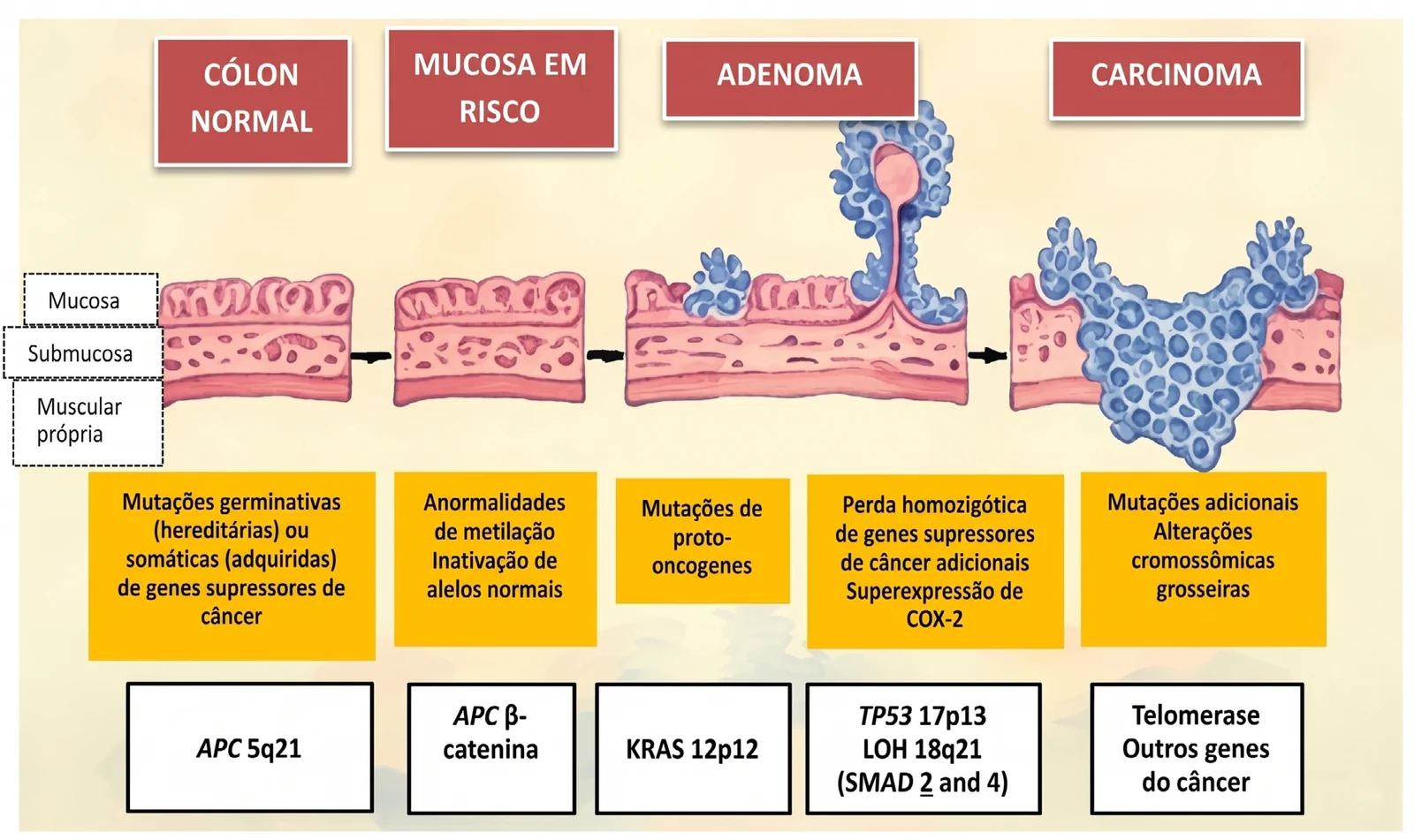
Morphological and molecular alterations according to the adenoma–carcinoma sequence.

Source: Adapted from Mitchell et al.^25^.

Regarding primary tumor location, 31 (32.30%) patients had tumors in the sigmoid colon, 26 (27.08%) in the rectum, 11 (11.45%) in the ascending colon, nine (9.40%) in the cecum, eight (8.30%) in the rectosigmoid junction, six (6.30%) in the descending colon, and five (5.20%) in the transverse colon. One patient presented with two synchronous primary tumors located in the ascending colon and rectum. In another patient (1.04%), the primary tumor site could not be determined because of extensive abdominal disease (Figure 2).

**Figure 2:**
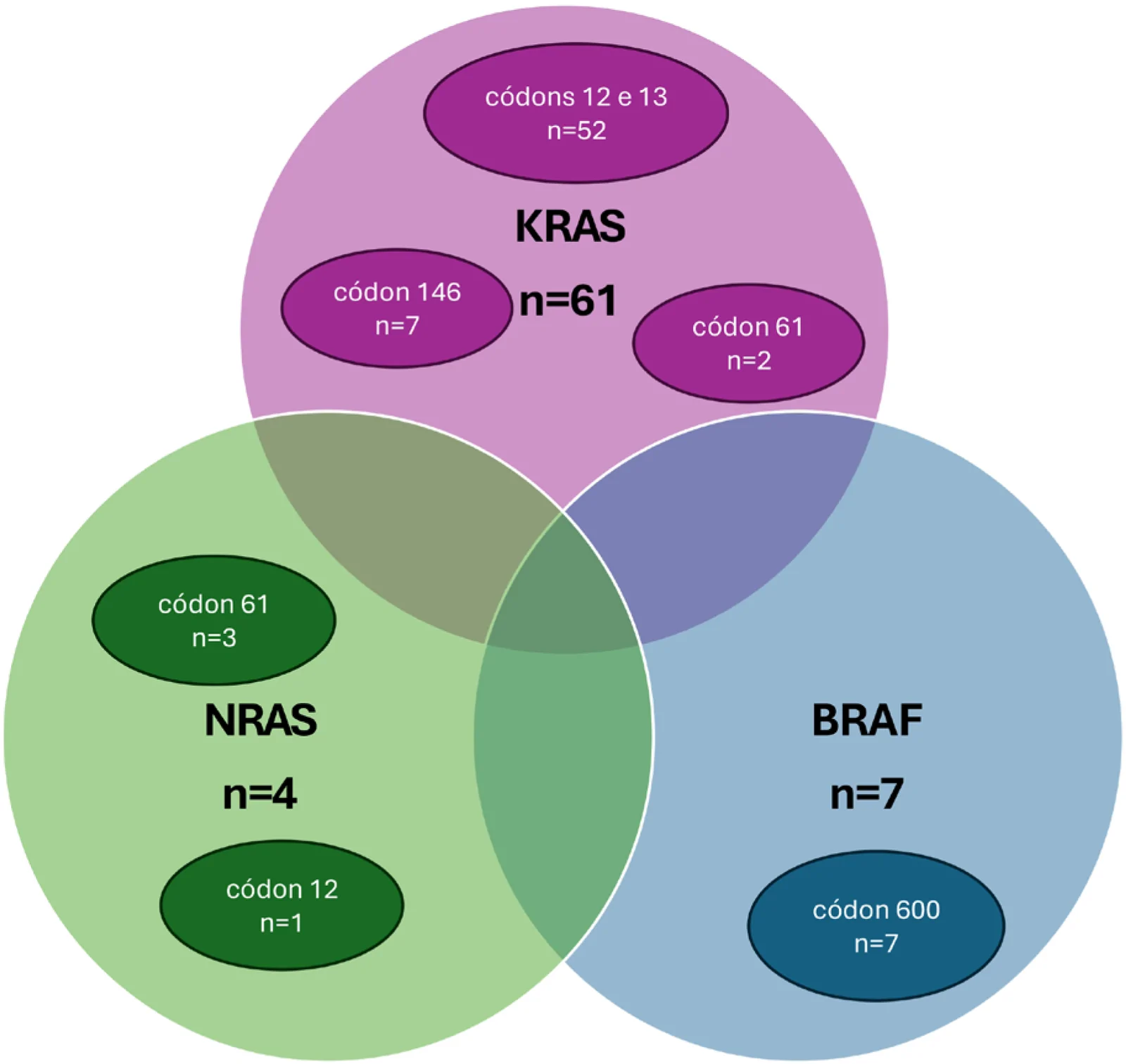
Distribution of single and concurrent mutations in the *KRAS*, *NRAS*, and *BRAF* genes.

The distribution of distant metastases was as follows: isolated liver metastases in 41 (42.70%) patients, isolated lung metastases in ten (10.40%), isolated peritoneal metastases in seven (7.30%), combined liver and lung metastases in 16 (16.70%), and metastases to other sites in 22 (22.91%). Metastatic disease was slightly more frequent among patients with *KRAS* mutations. As this study included patients treated at a single institution over a 1-year period, the generalizability of the findings may be limited (Table 1). The Figure 3 shows the Kaplan–Meier curve of survival probability of the patients studied.

**Table 1:** Analysis of the association between *KRAS* mutation and specific clinicopathological characteristics.

Clinicopathological characteristics		KRAS gene status		p-value
		Mutated	Wild-type	
Sex				
	Female	26	21	0.101
	Male	35	14	
Age group (years)				
	<60	20	9	0.468
	>60	41	26	
Tumor site				
	Cecum	4	5	0.275
	Ascending colon	6	5	
	Descending colon	6	0	
	Transverse colon	2	3	
	Sigmoid colon	21	10	
	Rectosigmoid junction	6	2	
	Rectum	17	9	
	Undetermined	0	1	
Tumor size (cm)				
	<2.5	0	2	0.210
	2.5–5.0	24	16	
	>5.0	18	7	
	Undetermined	19	10	
Pathological Stage				
	pT1	1	0	0.689
	pT2	4	1	
	pT3	21	16	
	pT4a	10	6	
	pT4b	8	2	
	Undetermined	17	10	
Metastatic Site				
	Liver	23	18	0.444
	Lung	7	3	
	Liver and lung	12	4	
	Peritoneum	5	2	
	Other organs	14	8	
CEA level (ng/mL)				
	<5	22	18	0.509
	5–100	23	9	
	100–500	12	6	
	>500	4	2	
Treatment				
	Chemotherapy	55	33	0.482
	Radiotherapy + chemotherapy	6	2	
Clinical status				
	Alive	41	28	0.180
	Deceased	20	7	

CA: carcinoembryonic antigen.

**Figure 3:**
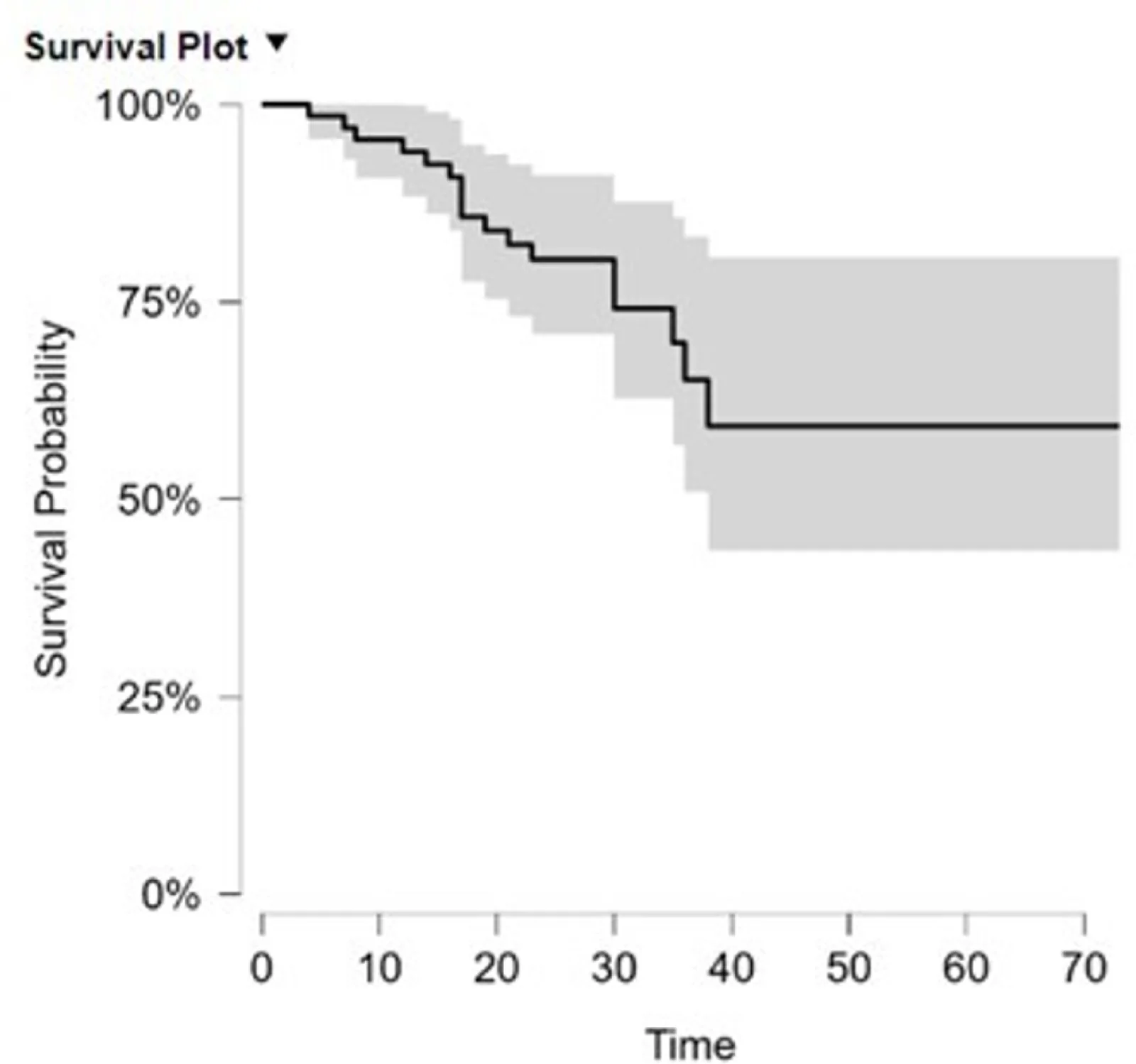
Kaplan–Meier curve.

## DISCUSSION

Mutations in the *KRAS* gene play a pivotal role in colorectal carcinogenesis and are associated with an increased risk of recurrence and mortality, as highlighted in several studies^4,6,26^. Evidence also suggests that *KRAS* mutations may represent an independent risk factor for the development of venous thromboembolism^9,12,13^, although further studies are required to clarify this association.

Most colorectal tumors arise from adenomatous polyps, which are benign lesions with the potential to undergo malignant transformation over time. Adenocarcinoma is the predominant histological subtype, accounting for approximately 95% of cases^3,14,15,18^. The *KRAS* gene encodes a small GTPase that functions as a molecular switch, regulating key cellular processes such as proliferation, differentiation, and survival. In its active, GTP-bound state, *KRAS* activates multiple intracellular signaling pathways, transmitting extracellular signals to the cell nucleus^20,22,25^.

Several studies have demonstrated that patients with CRC harboring *KRAS* mutations have shorter survival, a higher likelihood of developing metastases, and earlier disease recurrence than those with wild-type *KRAS* ^7,8,10,15,16^. *KRAS* mutations have been reported more frequently in right-sided colon tumors^33^.

Assessment of *KRAS* mutation status is now part of routine clinical practice for patients with metastatic CRC before initiating systemic treatment. Several laboratory methods are available for mutation detection using formalin-fixed paraffin-embedded tissue, fresh tissue, fine-needle aspiration specimens, or body fluids. Direct sequencing remains the gold standard^5,31,32^, although alternative techniques may provide greater sensitivity. The method of choice should consider both local availability and cost-effectiveness.

Determination of *KRAS* status is clinically relevant because these mutations are associated with poor prognosis and resistance to anti-epidermal growth factor receptor (EGFR) monoclonal antibodies, particularly cetuximab and panitumumab. Mutations affecting codons 12 and 13 of exon 2 confer resistance to these targeted therapies^19,24^. Therefore, all patients with metastatic CRC who are candidates for anti-EGFR therapy should undergo *KRAS* mutation testing before treatment.

Given the high mortality and therapeutic resistance associated with *KRAS*-mutated CRC, the development of more accurate diagnostic strategies and personalized therapeutic approaches has become increasingly important. This need is further reinforced by the substantial economic burden of CRC for health systems, including the costs of diagnosis, treatment, follow-up, and palliative care, as well as socioeconomic costs related to patient disability and reduced quality of life. Consequently, therapies tailored to patients’ molecular profile have the potential not only to improve clinical outcomes but also to reduce healthcare costs over the long term, positively impacting individual lives and the health system as a whole.

In the present study, *KRAS* mutations were identified in 63.54% of patients with metastatic CRC, a prevalence considerably higher than the 30 to 50% range generally reported in the literature^11,16,23,31^. Several factors may explain this discrepancy. First, our cohort was highly heterogeneous and consisted exclusively of patients with metastatic disease, who are more likely to harbor aggressive molecular profiles. Second, differences in the analytical sensitivity of mutation detection methods may have contributed to the higher mutation rate. Although direct sequencing is considered the gold standard, highly sensitive real-time PCR assays may identify mutations that would otherwise remain undetected. Finally, the high rate might reflect regional genetic and environmental characteristics of the population in the southernmost part of Brazil, although population-based genomic studies are needed to confirm this hypothesis.

Whereas previous studies have consistently associated *KRAS* mutations with right-sided (proximal) colorectal tumors^28^, our findings demonstrated a predominance of left-sided tumors, particularly in the sigmoid colon. This observation challenges the conventional paradigm of tumor laterality and may reflect regional genetic characteristics or interactions with other molecular alterations. However, no significant association was observed between *KRAS* mutation status and patient sex or age, in agreement with previous reports^20^. The relatively small sample size (n=96) might have limited the statistical power to detect more subtle associations. Moreover, the retrospective design of the study relies on the accuracy of hospital records.

Overall survival was similar between patients harboring codon 12 and codon 13 mutations, consistent with previous findings^7^. These results suggest that the presence of a *KRAS* mutation, regardless of the affected codon, is associated with an unfavorable prognosis. By contrast, Ahn et al.^1^ reported that codon 13 mutations may have less prognostic significance than codon 12 mutations in patients with CRC.

Li et al.^13^ found that codon 12 mutations are associated with lymph node metastasis and more advanced tumor stage compared with tumors lacking these mutations. Kassaw et al.^12^ conducted a systematic review including 5,045 patients with CRC from 14 African countries, based on studies published between 2009 and 2025. The pooled prevalence of *KRAS* mutations was 39%, with exon 2 mutations accounting for approximately 37% of cases. Two of the included studies also found a significant association between *KRAS* mutations and the mucinous adenocarcinoma subtype (p<0.05). Cortes-Torres et al.^8^ evaluated the clinicopathological characteristics of 51 patients with *KRAS*-mutated CRC in an analytical cross-sectional study. Variables included sex, age, tumor location, *KRAS* and *BRAF* mutations, and metastasis status. The sigmoid colon was the most frequent primary tumor location (35.3%), consistent with our findings. *KRAS* mutations were detected in 35% of patients, with codon 12 mutations being the most prevalent.

Amirifard et al.^3^ investigated patients with metastatic CRC treated at Imam Reza Hospital, Kermanshah Province, Iran, between 2012 and 2016. The mean age at diagnosis was 51.5±12.6 years (range of 22 to 76 years). No significant association was observed between *KRAS* mutation status and clinicopathological characteristics.

Another aspect that has been explored in the literature is the relationship between *KRAS* mutations and depression, which was not investigated in our study. Zhou et al.^32^ evaluated 62 patients with metastatic CRC and found that 28 were positive for probable depression. The prevalence of *KRAS* mutations was 40.3% (25/62), and 76.0% of patients with *KRAS* mutations had probable depression compared with only 24.3% of those with wild-type *KRAS* (p<0.05). These findings suggest a possible relationship between *KRAS* mutation status and depression that warrants further investigation in a larger and more representative sample.

## CONCLUSIONS

*KRAS* mutations were identified in more than half of the patients, representing a prevalence higher than that generally reported in the literature. This finding may be explained by the high heterogeneity among study populations, especially given the inclusion of patients with metastatic disease and more aggressive clinical characteristics.

Contrary to the well-established association between *KRAS* mutations and right-sided colorectal tumors, our cohort showed a predominance of left-sided tumors, particularly in the sigmoid colon. This unexpected finding may reflect genetic and environmental peculiarities of the *Gaucho* population or interactions with other molecular alterations and should be validated in larger, independent cohorts.

The presence of *KRAS* mutations is associated with a poorer prognosis, including increased metastatic potential and reduced response to conventional therapies, resulting in worse overall and disease-free survival. Although some of our findings differ from those previously reported, the present study supports an association between *KRAS* mutation status, clinicopathological characteristics, and tumor anatomical distribution in metastatic CRC.

Determination of *KRAS* mutation status is essential for guiding therapeutic decision-making in patients with metastatic CRC, potentially contributing to increased survival. Nevertheless, access to molecular testing remains limited for many patients, especially in resource-constrained settings, underscoring the need to expand the availability of this important diagnostic tool.

## Data Availability

The datasets generated and/or analyzed during the current study are available from the corresponding author upon reasonable request.
